# Impact of self-directed learning strategy, an innovative method in nursing undergraduates: Study protocol for a randomized controlled trial

**DOI:** 10.1371/journal.pone.0325300

**Published:** 2025-07-07

**Authors:** Sabina Chaudhary, Adarsh Lata Singh

**Affiliations:** 1 Department of Nursing, Unique Medical College & Teaching Hospital, Rajbiraj, Saptari, Nepal; 2 Department of Dermatology, Jawaharlal Nehru Medical College, DMIHER, Wardha, Maharashtra, India; University of Sharjah College of Health Sciences, UNITED ARAB EMIRATES

## Abstract

**Background:**

In today’s fast-paced healthcare environment, self-directed learning is essential for healthcare professionals to stay updated and provide optimal care. Game based learning has a potential of motivating students’ engagement and creating fascinating self-directed learning environment for favorable outcome. This randomized controlled trial aims to investigate the impact of game-based learning as an innovative self-directed learning strategy compared to conventional self-directed learning strategy on knowledge acquisition, and self-directed learning abilities among nursing undergraduates based on Self-directed learning Instrument (SDLI) score.

**Methods:**

This quantitative, randomized controlled trial will enroll and randomize 140 undergraduate nursing students in the experimental and control group using stratified random sampling and follow them for 12 weeks. Self-directed learning (SDL) orientation session will be conducted prior to the randomization for all the participants. After randomization, experimental group will undergo SDL with game-based learning and control group will undergo SDL with conventional learning for four weeks. Follow up sessions will be conducted once a week for a period of 4 weeks. Evaluation of impact of intervention will be assessed at four time points: preintervention, immediately postintervention, 4 weeks postintervention and 12 weeks postintervention using structured knowledge questionnaire, Self-Directed Learning Instrument (SDLI) and Cognitive, Affective and Psychomotor (CAP) perceived learning scale. ANOVA will be used to compare the variations in self-directed learning ability, perceived learning competency, and knowledge between two groups. Regression analysis will be conducted to explore the correlation between various independent variables and the dependent variables. Paired t-test will be used to analyze the variation between pre-test and post-test results, considering a p-value less than 0.05 to be statistically significant.

**Discussion:**

This trial aims to determine whether game-based learning as an innovative self -directed learning strategy is superior to conventional self-directed learning strategy in improving knowledge level and self-directed learning abilities of nursing undergraduates. This trial is approved by the Datta Megha Institute of Higher Education & Research, Institutional ethics committee (DMIHER(DU)/IEC/2023/141C). We plan to disseminate study results in peer-reviewed journals and international conferences.

**Trial registration:**

Trial Registered Prospectively [CTRI/2024/01/061599].

## Introduction

### Background

Nurses play a crucial role in healthcare worldwide, and with advancements in technology and new health demands, the nursing profession is evolving. To adapt to these challenges, nursing students need to strengthen their self-directed learning abilities [1]. Incorporating self-directed learning in nursing education promotes critical thinking, lifelong learning, and professional development [2–4]. This approach allows students to remain flexible, proficient, and resourceful in meeting the ever-changing healthcare needs of society and medical knowledge [5,6]. By being a self-directed learner, nursing students can achieve academic success and foster a habit of staying updated with healthcare modalities to provide safe patient care [7,8].

Self-directed learning (SDL) is a learning approach where students take responsibility for their learning process, set goals, and identify resources for knowledge acquisition [9]. However, some nursing students may struggle due to factors like prior educational experiences, learning preferences, and confidence levels [8]. A systematic review and meta-analysis by Nazarianpirdosti M revealed that the level of SDL in nursing students is far from desired levels, suggesting further studies to evaluate the impact of various strategies and factors facilitating and inhibiting SDL [10]. Similarly, a cross-sectional study by Li-Qing Tang et.al found low SDL ability in nursing undergraduates [1].

According to Altmiller and Pepe (2022), Traditional teaching methods in nursing education, such as lectures and modules, have proven successful but may not adequately train students to be self-directed, critical thinkers, and problem solvers [11]. Therefore, the growing emphasis on lifelong learning in the nursing profession necessitates educators to equip students with effective self-directed learning competencies. Traditional self-directed learning approaches, such as identifying learning needs, setting goals, and regulating learning, have some merit. However, there is a growing demand for novel approaches that can enhance the self-directed learning experience in nursing undergraduates. Studies recommends that nursing educators can improve students’ self-directed learning ability by changing their learning environment and incorporating modern technology. Since motivation is crucial for engagement and self-directed learning, the strategies that promote student engagement and create a stimulating learning environment are essential. Nursing institutions worldwide are embracing technology-driven self-directed learning approaches to enhance educational outcomes and equip students with the competencies needed to manage real-world healthcare challenges. Nursing programs are integrating self-directed learning into their curricula through interactive online modules, high-fidelity simulation-based learning experiences, flipped classrooms, blended learning environments, virtual and augmented reality training environments, problem-based learning, and competency-based learning.

Self-directed learning (SDL) strategies vary greatly across institutions and countries, influenced by factors like institution culture, faculty expertise, resources, and regulations. Developed countries often adopt technology-based SDL strategies because of their improved technological infrastructure, greater financial resources, and enhanced experimental culture in education. However, in developing countries, despite the recognition of SDL as a key to improving nursing education quality, challenges such as limited access to advanced technologies, insufficient internet connectivity, and the widespread use of traditional teaching practices hinder its adoption.

Although innovative technology-based strategies are being incorporated in nursing education programs, their potential in improving self-directed learning abilities in nursing students compared to conventional method is still unclear. This makes it difficult to make informed decisions on choosing the finest and most reliable teaching learning methodology for enhancing lifelong learning abilities while designing and implementing curriculum on the basis of the available research. This highlights the need for evaluative research on innovative SDL strategies that can be effectively implemented in both face-to-face and virtual learning environments, thereby enhancing the quality of nursing education.

At present, game-based learning is a growing trend in health profession education, enhancing student experience and improving study outcomes. So, it can be one of the potential technology-based approaches for self-directed learning as it has proven effective in engaging learners, promoting active participation, and improving knowledge retention. A systematic literature review by Tavares N. (2022), Kuruca Ozdemir E et.al (2022), & Xu Y. et al. (2021) highlights the positive impact of game-based learning on nursing undergraduate students’ learning experience and knowledge retention [12–14]. The findings support the integration of game-based learning strategies in nursing education, but further research is needed to explore the long-term effects of game-based learning and its impact on affective and behavioral learning outcomes.

Interestingly, over the recent years, mounting evidence suggests that game-based learning has proven to be more efficient than traditional teaching methods as it provides an interactive and engaging environment that promotes intrinsic motivation, autonomy, immediate feedback, progress tracking, scaffolded challenges, and personalization enabling self-directed learning. It empowers learners to take control of their learning, make decisions, and actively engage in the learning process [12–18]. This approach could be beneficial for nursing education, but its impact on self-directed learning abilities has been under-examined [19].

This fact demands research to address this gap and contribute to the growing body of evidence focused on whether the game-based learning is equally effective for nursing students to enhance their self-directed learning abilities. Overall, the drive to conduct research in this area has stem from a combination of pedagogical challenges in nursing education, the potential of game-based learning to address those challenges, and the need for more empirical evidence to support the integration of innovative learning strategies like game-based learning in nursing curricula. The aim of this research is to investigate if game-based learning can enhance self-directed learning and information retention, helping tailor educational approaches to diverse learners and better understand their suitability and potential challenges.

This research can contribute to prevailing evidence that create a strong connection between game-based learning and self-directed learning abilities of nursing students, which will provide a solid foundation for empirical decision-making in curriculum design and educational policy, ensuring that nursing students are equipped with the knowledge, skills, and self-directed learning abilities necessary to excel in their future careers. Furthermore, the findings might suggest the need for development of more comprehensive and personalized game-based learning tools facilitating self-directed learning abilities and helping nursing students to meet the unique challenges and needs of the profession.

The proposed framework for self-directed learning in this study is developed by mapping the psychological constructs of self-determination theory (SDT) [20] and elements of gaming into the paradigm that the autonomous motivation for initiation of self-directed learning and favorable learning outcome can be fostered by satisfying the psychological needs of autonomy, competence, and relatedness through gamification. Gamification is a transformative approach to education, combining games and learning to engage the current generation of young and technology-savvy learners. By integrating gaming elements with self-determination theory and Garrison’s self-directed learning model [21], educators can create a more engaging, motivating, and effective learning environment that encourages learners to take charge of their educational journey and promote self-directed learning.

**Fig 1:**
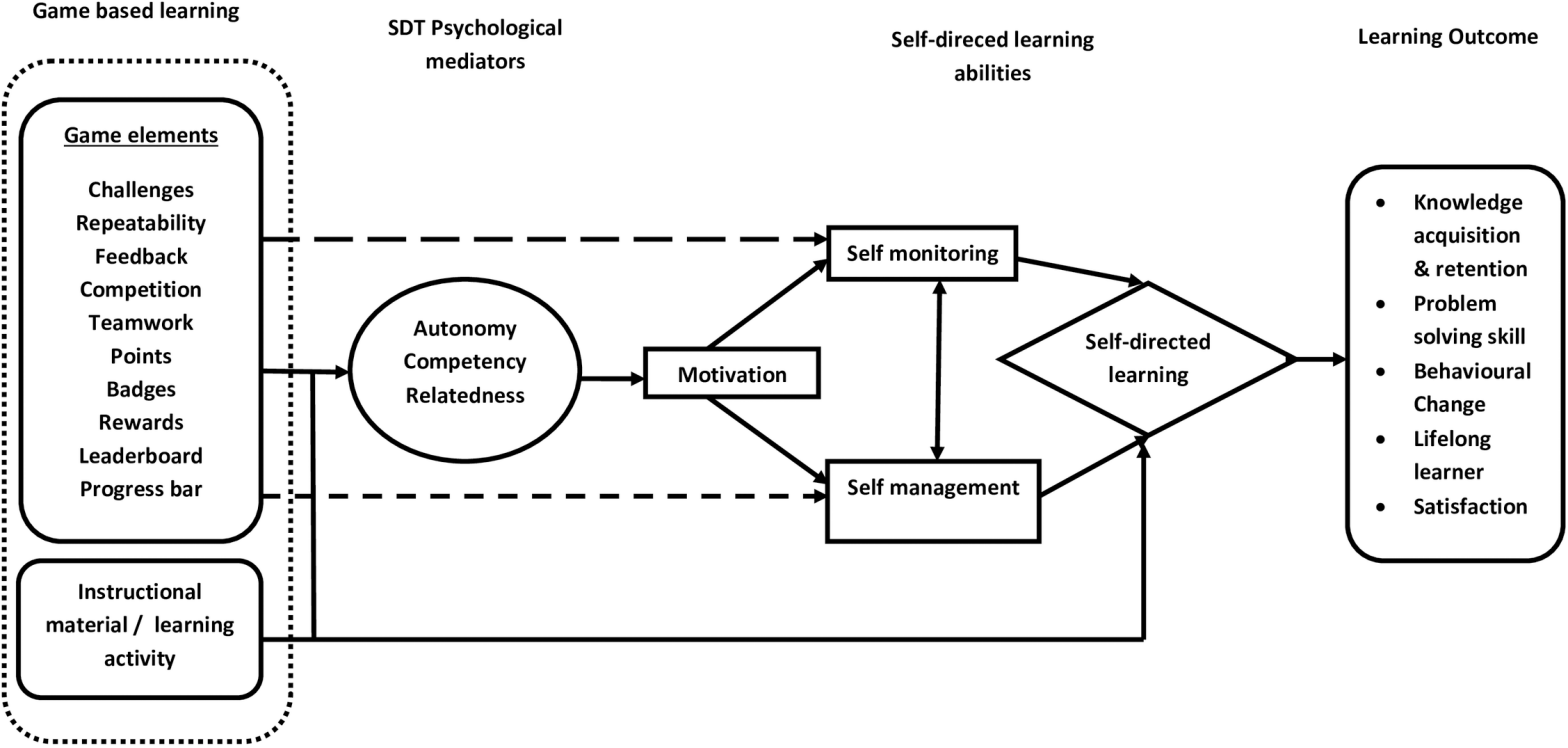
Conceptual framework explaining impact of game-based learning on self-directed learning adopted from Landers’ theory of gamified learning (2014), Self-determinant theory and Garrison’s model of self-directed learning.

Fig 1 illustrates this framework. Gaming elements can satisfy the psychological needs of SDT by providing learners with a sense of control, and opportunities to develop mastery and social connections through collaborative and competitive activities. The framework proposes that if participating in a learning activity integrated with gaming elements provides the learner with a sense of autonomy and competence as well as a sense of belonging, then motivation would be enhanced and learners are more likely to engage deeply and persistently with their learning tasks resulting in knowledge retention, problem-solving skill and improvement in overall academic performance.

### Objective

This study aims to compare the effect of the game-based learning as an innovative self-directed learning strategy with conventional self-directed learning strategy on knowledge acquisition, and self-directed learning abilities in nursing undergraduates through a randomized controlled trial.

### Hypothesis

H_1_: The incorporation of game-based learning as an innovative self-directed learning strategy will be superior to conventional learning method in improving self-directed learning abilities among nursing undergraduates.

H_2_: Nursing students in game-based learning group will have higher knowledge scores than those in conventional learning group.

## Methods

### Trial design

**Fig 2:**
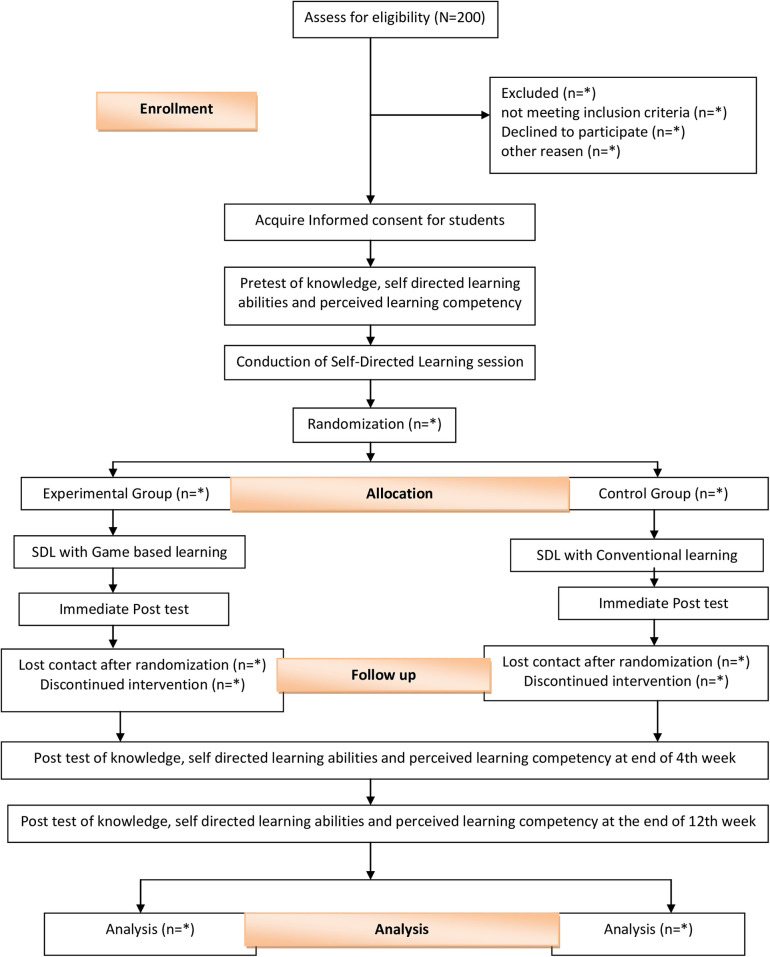
Schematic diagram of trial.

We have designed a prospective, single centric randomized controlled trial with data analyst-blinded and two parallel groups allocated at a 1:1 ratio. Study period will be 12 weeks, where participants will be followed for first 4 weeks and will be assessed at four points of time, i.e., preintervention, immediately postintervention, 4weeks postintervention and 12 weeks postintervention to identify the impact of game-based learning as an innovative self-directed learning strategy compared to conventional self-directed learning method among nursing undergraduates. A total of 140 undergraduate nursing students will be enrolled and assigned to either experimental group (SDL with game-based learning) or control group (SDL with conventional learning) through stratified random sampling. A schematic diagram presenting enrolment, random allocation, intervention, follow-up and data analysis of the study is shown in Fig 2

**Fig 3:**
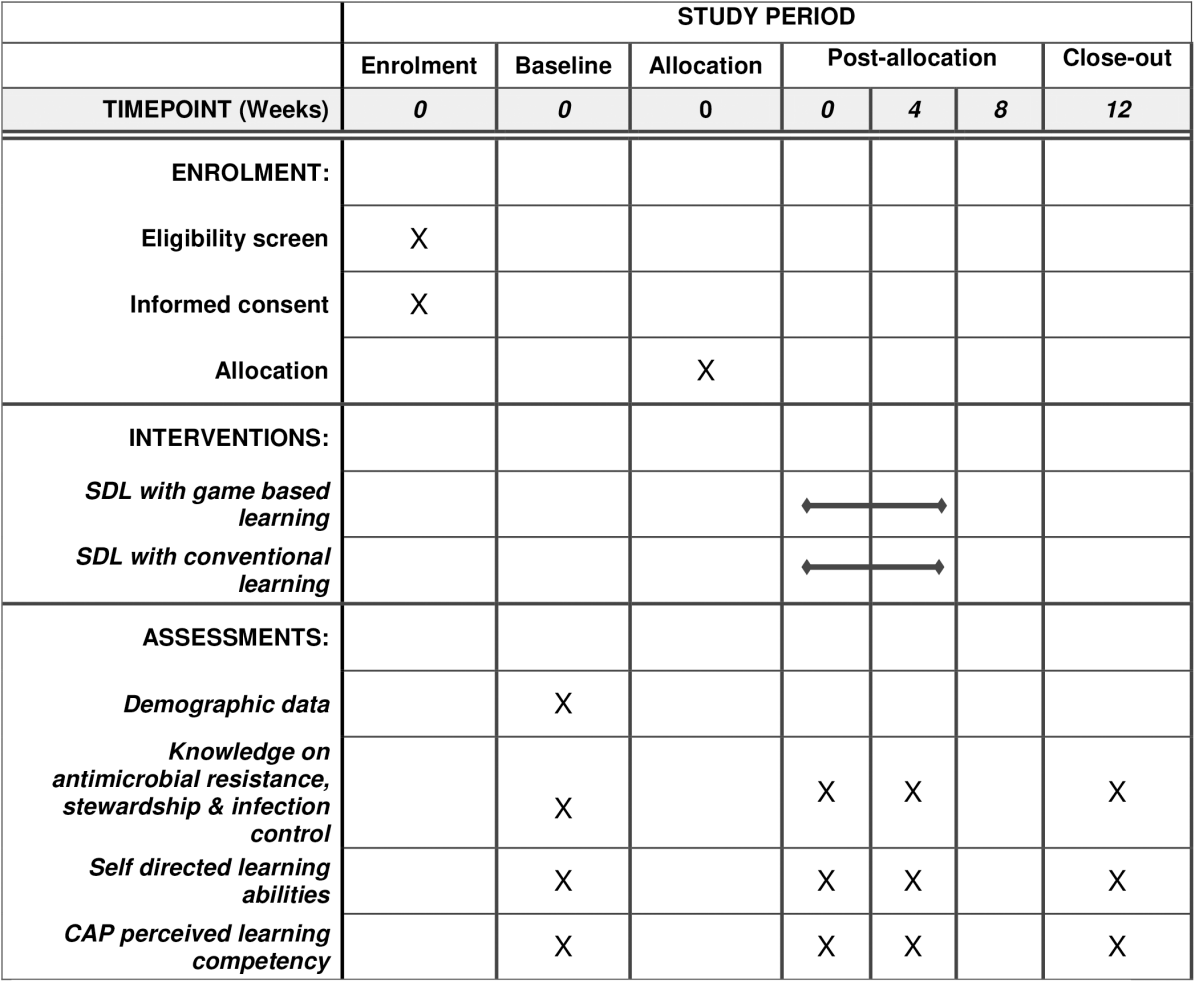
Schedule of enrolment, intervention and assessment for study (SPIRIT figure).

The study timeline and schedule of enrollment, interventions and assessments (Standard Protocol Items: Recommendations for Interventional Trials (SPIRIT) figure) and the SPIRIT Checklist are provided as Fig 3. and S1 File, respectively.

### Setting

This trial will be conducted at Smt. Radhikabai Meghe Memorial College of Nursing (SRMMCON), a renowned nursing college located in Sawangi (Meghe), Wardha, Maharashtra, India. SRMMCON is affiliated with the Datta Meghe Institute of Higher Education & Research (Deemed to be University) and is recognized by the Indian Nursing Council (INC) and the Maharashtra Nursing Council (MNC). It is dedicated to providing high-quality nursing education and training and offers undergraduate, postgraduate, postgraduate diplomas and doctoral program in nursing.

### Eligibility criteria

#### Inclusion criteria.

1. Participants must have been enrolled in an accredited undergraduate nursing program (B.Sc. Nursing).
2. Participants in their first or second year of study, who are most likely equipped with basic nursing knowledge but are still improving their clinical skills and self-directed learning (SDL) routines.
3. Participants who give informed consent and show a willingness to take part in the study.
4. Participants should have basic proficiency in English language, as SDL materials and assessments is in English language.
5. Participants must have access to a computer or laptop or tablet or smart phone, and internet connectivity, as SDL strategies rely on online resources and e-learning platforms.

#### Exclusion criteria.

1. Students who have received previous classes or training in antimicrobial resistance, antimicrobial stewardship and infection control should be excluded to prevent any potential influence on the intervention’s outcomes.
2. Students who have a record of irregular attendance in class, as SDL requires dedication and regularity.
3. Students who continuously fail to engage in intervention or express a desire to withdraw can be removed from the study.

### Interventions

The researcher will provide participants with detailed information about the study’s purpose and procedures, ensuring anonymity of personal information and collected data. Participants will be informed about the questionnaire’s time and can withdraw at any time. Written informed consent will be obtained from willing participants. After signing an informed consent form, participants will undergo a pretest on knowledge, self-directed learning abilities, and CAP perceived learning competency. Students from both the semesters will participate in a 90 minutes structured teaching session on self-directed learning, discussing its concept, importance and process. Subsequently, participants will be randomly assigned to either the intervention or control groups.

#### In experimental group.

The experimental group will be divided into small teams and exposed to the Antimicrobial Stewardship (AMS) game, developed by the Commonwealth Partnerships for Antimicrobial Stewardship Programme (CwPAMS*) and Focus Games Ltd. The game teaches the basics of Anti-microbial resistance, stewardship, and infection control, and their impact on global health. The game encourages group discussion and challenges players to change behaviors that can save lives. After the gaming session, the experimental group will undergo an immediate postintervention test, and will be provided with login details for the next online AMS gaming session. Four gaming sessions will be conducted over four weeks.

#### In control group.

The control group will be provided with information booklet on antimicrobial resistance, antimicrobial stewardship and infection control and assign for self-learning for one hour. Immediate post-test will be taken after one hour of self-study and encourage to do self-learning with provided learning material for four weeks.

### Strategies to improve adherence to interventions

To improve adherence to intervention protocol, text message in WhatsApp and email will be send to all the participants. The researcher will interact with participants weekly for one month on a Zoom online platform to provide guidance on self-directed learning and answer questions about learning methods. However, no lectures will be given on the learning content so that outcome variables are not affected.

To promote participant retention and minimize the risk of communication between participants in the control and experimental groups during the 4-week of follow-up period, we will implement several strategies. Participants will be instructed on maintaining confidentiality of group assignments and intervention details. The control group will be assured of getting access to the AMS game at the end of the study to reduce tendency for seeking premature information. Only experimental group will get login code for zoom platform for playing AMS game, ensuring only the assigned group can attend each session. During the game-based learning session, all participants will be asked to keep their cameras on to ensure that only the experimental group participants are present.

### Outcomes

The outcome measurements will be conducted at baseline, immediately postintervention, 4 weeks postintervention, and 12 weeks postintervention using the same data collection tool.

#### Primary outcome.

*1. Self-Directed Learning abilities Improvement:* The primary outcome would be improvement in self-directed learning (SDL) abilities among nursing undergraduates after intervention. This will be measured by using validated SDLI tool, which assess key SDL competencies such as:

a. Learning motivation
b. Planning and implementing
c. Self-monitoring and
d. Interpersonal communication

The difference in Pre- and Post- intervention SDLI scores between the experimental and control groups would highlight the effectiveness of game-based learning in improving SDL abilities.

*2. Knowledge Acquisition:* Another primary outcome is improvement in knowledge acquisition in the topic antimicrobial resistance, antimicrobial stewardship & infection control. This will be assessed by using structured questionnaire developed and validated by researcher. The difference in scores between the game-based learning and conventional SDL group would highlight the effectiveness of each strategy in improving knowledge acquisition and retention.

#### Secondary outcome.

*1. Cognitive, Affective and Psychomotor perceived learning Improvement:* Improvement in the cognitive, affective and psychomotor domain of CAP perceived learning competency scale on postintervention score of experimental group will signify positive impact of intervention.

### Sample size

The study uses G*Power (version 3.1.9.7) to determine the sample size. Assuming a significance level of 0.05 and effect size of 0.5 based on meta-analysis study by Bai, S., Hew, K.F., & Huang, B. (2020) [22], which estimated a medium effect size in favor of game-based learning over learning without gamification, 64 participants per arm is required for a statistical power of 80%. Considering a dropout rate of 10%, 70 participants per arm, totaling 140 participants is required for study.

### Recruitment

The accessible population for this study is undergraduate nursing students at Smt. Radhikabai Meghe Memorial College of Nursing (SRMMCON), specifically first, second, and third semester students. The total accessible population consists of 200 students, with 100 students in each first and third semester. To ensure equal representation, stratified random sampling will be used. The population will be divided into strata based on semester and 70 students will be randomly selected from each stratum. Each eligible student will be assigned a unique identification number. The study will select 70 students from the first semester students’ list and another 70 from the third semester students’ list using a random number generator.

### Randomization and allocation concealment

The study involves randomly allocating 140 students to either an intervention group (SDL with game-based learning) or a control group (SDL with conventional learning), stratified based on semester to ensure equal distribution of first-semester and third-semester students. Within each semester, 70 students will be split equally between the intervention and control groups using a random number generator. Independent personnel not involved in the study will use random number generator software to generate a random number, allocating subjects into two groups in 1:1 ratio. Random numbers will be placed in coded, opaque envelopes and entrusted to personnel not involved in the study until the random allocation and intervention are initiated.

### Blinding

An independent assessor, not involved in intervention delivery will conduct outcome assessment for an educational intervention. Due to the nature of the intervention, blinding the researcher is not possible. The data analyst will be blinded in the study. The data will be coded with neutral labels, ensuring unbiased analysis without revealing group assignments.

### Data collection

The researcher will use a structured and pre-tested data collection inventory to gather study data. The data will be entered into the study database promptly. After obtaining written consent, a self-administered inventory will be distributed and collected from students. Data collection will occur at four time points: preintervention, immediately postintervention, 4 weeks postintervention, and 12 weeks postintervention using the same inventory.

Data collection inventory comprises of four sections:

A. **Socio-demographic information:** age, gender, semester, place of residence and previous exposure to game-based learning.
B. **Knowledge questionnaire:** A 15-item multiple-choice questionnaire on antimicrobial resistance, antimicrobial stewardship, and infection control will be used to assess knowledge. The score ranges from 0–15, with scores categorized as adequate knowledge (above 75%), moderately adequate knowledge (between 51–75%), and inadequate knowledge (less than 50%). Each item will be tested for validity and reliability before the study.
Expert evaluation will confirm content validity, with a panel of subject matter experts assessing relevance, clarity, and alignment with study objectives. Cronbach’s alpha will be used to determine internal consistency, with a Cronbach’s alpha value of 0.70 or higher indicating acceptable reliability. Test-retest reliability will be assessed by administering the questionnaire to a sample of participants at two different time points (with an interval of two weeks). Intraclass correlation coefficients (ICC) will be used to assess results’ consistency over time, with an ICC value greater than 0.75 indicating strong reliability.
C. **Self-Directed Learning Instrument (SDLI)** [23]: The Self-Directed Learning Instrument (SDLI) is a reliable tool developed by Cheng et al. (2010) with good internal consistency (Cronbach’s total scale was α = 0.916). Cadorin L et al. (2017) recommend SDLI for assessing SDL abilities among nursing students and nurses due to its excellent methodology quality, Cronbach α ranging from 0.73 to 0.91, and structural validities. Content validity was supported by two rounds of Delphi study. It is comprised of 20 items categorized into four domains:
  a. Learning motivation: six items (α = 0.80),
  b. Planning and implementing: six items (α = 0.86),
  c. Self-monitoring: four items (α = 0.78), and
  d. Interpersonal communication: four items (α = 0.76).
The Self-directed learning instrument (SDLI) metric uses a five-point Likert scale to measure self-assessment of self-directed learning (SDL) abilities. Scores range from 1 to 5, with “strongly disagree” indicating very low level of (self-assessed) abilities, and “strongly agree” indicating high level of (self-assessed) SDL abilities. All the statements are stated positively and the total possible score ranges from 20 to 100, with higher scores indicating higher SDL abilities.
D. **Cognitive, Affective & Psychomotor (CAP) perceived learning scale** [24]: The study will utilize the CAP Perceived Learning Scale, a nine-item, 6-point Likert scale, developed by Rovai et al. (2009), to measure perceived cognitive, affective, and psychomotor learning in game-based and conventional learning environments. The scale is a reliable and valid tool with internal consistency of 0.79 and supported concurrent validity. It generates an overall score representing perceived learning across three domains: cognitive learning, affective learning, and psychomotor learning.

Subscale cognitive learning describes a students’ ability to recall or recognition of knowledge, affective learning is the positive attitude towards the subject matter, and psychomotor learning is the capacity to perform tasks (Rovai et al., 2009). In CAP Perceived Learning Scale, statements 1,2 and 5 measures perceived cognitive learning; statement 4,6, and 9 measures perceived affective learning and statement 3, 7, and 8 measures perceived psychomotor learning. Statement two and seven are inversely scored while the other statements are scored in a standard pattern. Total scores can range from a maximum of 54 to a minimum of 0 and CAP subscale scores can range from maximum of 18 to minimum of 0. A high CAP Perceived Learning Scale score is an indicator of a keen perception of learning by students participating in the study (Rovai et al., 2009).

Self-directed learning instrument (SDLI) and CAP Perceived learning Scale will be used for the study in its original form without any modification. Participant information with personal identifiers will be stored in paper-based form in a locked filing cabinet in a secure and locked private space. To ensure accuracy and completeness, study data will be reviewed by the researcher.

### Strategies for promoting participant retention, ensuring complete follow-up and handling deviation from study

The researcher will follow up with participants who miss sessions or show non-compliance, offering support and solutions if needed via email, WhatsApp or phone call. Participants with workload, technological issues, or personal difficulties will be offered solutions (e.g., flexibility in gaming session or SDL discussion session, technical support). Participants who deviate but do not withdraw will be included in an intention-to-treat analysis to preserve randomization integrity. Those who fail to engage or express a desire to withdraw will be removed from the study and documented with reason of withdrawal for proper interpretation of study result and impact on sample size. If attrition is high, additional participants will be randomized to fill gaps and maintain the study’s statistical power.

### Data management and monitoring

The study will use SPSS software for data management, including entry, validation, coding, cleaning, and preparing the final database. The researcher will maintain and store study records, including informed consent. The Data Monitoring Committee (DMC) is not essential due to nature, scale, and resources availability. Alternative monitoring measures will be implemented to ensure scientific and ethical integrity. Data management will comply with institutional ethics committee for participant protection and confidentiality. Regular review of trial progress will ensure data quality and accuracy.

### Statistical analysis

The study will use SPSS version 21, with tests like Shapiro-wilk, skewness, and kurtosis to evaluate the normal distribution of variables. Pre-test homogeneity will be assessed using descriptive statistics, independent t-tests, chi-square tests, and Fisher’s exact test. ANOVA will compare differences in SDL ability, PL competency, and knowledge between groups. Regression analysis will explore the relationship between independent variables and dependent outcomes. Differences between pre-test and post-test will be analyzed using paired t-test. All statistical analysis will be two-sided, with a p-value less than 0.05 considered statistically significant.

### Ethical consideration

The study will follow the Declaration of Helsinki 2008 and has been approved by the Institutional ethics committee (IEC) of Datta Megha Institute of Higher Education & Research. Participants will be informed about the study’s purpose and procedures, and their personal information and collected data will be kept anonymous. The questionnaire will take around 15–25 minutes, and participants can withdraw at any time. Written informed consent will be obtained from those willing to participate. A unique alpha-numeric identifier will be assigned to each participant to safeguard confidentiality.

To ensure fairness, participants in the control group will be granted access to the AMS game after a 12th week follow-up post-test. They will be guided on navigating the game, understanding its objectives, and effectively using its features to maximize learning outcomes. The control group will not only have access to the AMS game but also understand how to use it effectively to achieve the same educational benefits as the intervention group.

## Discussion

### Dissemination plan

We plan to disseminate study results in peer-reviewed scientific journals and academic conferences, targeting educators and practitioners, students, academic researchers, educational organizations and policy makers, thereby fostering the widespread sharing of scientific knowledge and the advancement of best practices.

### Limitations

This study has some limitations. Results obtained from a single center may not be representative of the broader population of nursing undergraduates and limit the generalizability of the findings. The inventory is self-reported, and self-reported outcomes may introduce a range of bias such as data credibility. A 12-week study period may not be sufficient to capture long-term impacts or changes in learning outcomes. There is no assurance that participants of control group and experimental group will not communicate in spite of various strategies to avoid contamination as they are classmates and spend maximum time together.

### Safety

This randomized trial aims to explore the effectiveness of game-based learning as an innovative self-directed learning strategy compared to conventional methods. While games can be engaging, they may lead to overemphasis on game mechanics rather than educational content. This could demotivate students who don’t enjoy gaming or find it irrelevant to their academic pursuits, reducing learning outcomes. To mitigate this risk, the game content chosen for this trial is academically rigorous, relevant to nursing practice, and aligned with the nursing outcome-based curriculum and evidence-based guidelines. Clear guidelines and expectations shall be communicated to participants. Technical glitches, internet connectivity issues, or hardware malfunctions can disrupt the learning process. To ensure smooth gaming and learning, regular communication via WhatsApp and prompt technical support will be provided.

## Supporting information

S1 File SPIRIT 2013 checklist. (PDF)

S2 File Research protocol. (PDF)
