# Supplementary material for: Impact of self-directed learning strategy, an innovative method in nursing undergraduates: Study protocol for a randomized controlled trial
Source: PLoS One. 2025 Jul 7;20(7):e0325300. doi: 10.1371/journal.pone.0325300 (PMC12233217; doi:10.1371/journal.pone.0325300)
Supplement: S2 File — (PDF) [file pone.0325300.s002.pdf]

Impact of Self-Directed Learning strategy, an innovative method in nursing undergraduates: A randomized controlled trial

A
SYNOPSIS
SUBMITTED TO THE
DATTA MEGHA INSTITUTE OF HIGHER EDUCATION & RESEARCH
FACULTY OF INTERDISCIPLINARY SCIENCE

FOR THE DEGREE OF
DOCTOR OF PHILOSOPHY
IN
HEALTH PROFESSION EDUCATION

BY

SABINA CHAUDHARY

UNDER THE GUIDANCE OF

DR. ADARSH LATA SINGH

Director

School for Health Professions Education and Research

Datta Meghe Institute of Higher Education & Research (Deemed to be University), Wardha

2022

Table of Contents

Title of the study 1
Introduction 1
Background and rationale 1
Literature review 4
Research gap analysis 7
research questions 11
Hypothesis 11
Aim 11
Objectives 11
Methods 12
Study design 12
Participants, interventions, and outcomes 12
Study setting 12
Eligibility criteria 12
Sample size 13
Recruitment 13
Assignment of interventions (for controlled trials) 14
Allocation 14
Blinding (masking) 14
Interventions 14
Outcomes 17
Participant timeline 17
Data collection, management, and analysis 19
Data collection methods 19
Data Management & Monitoring 20
Statistical methods 21
Translatory component (conceptualized) 21
Ethics and dissemination 21
IEC approval 21
Consent 21
Confidentiality 21
Dissemination plan 22
References 22
Appendices 26
Gantt chart for phd research 26

RESEARCH PROTOCOL

Title of the Study

Impact of Self-Directed Learning strategy, an innovative method in nursing undergraduates: A randomized controlled trial.

Introduction

Background and Rationale

Globally, nurses are known to play a crucial role in rendering health care services and with the progression of time, advancement in health care technology and emergence of newer health demands and chronic diseases, the scope of nursing profession is changing drastically. To face today's healthcare challenges confidently, nursing students should receive high-quality education and training through an improved educational system that will support seamless academic progression and prepare nursing graduates to meet a variety of patient needs. Nursing students should also take the initiative to advocate for change and make efforts to advance nursing science that benefits patients and the ability of the health profession to provide safe and high-quality patient care.(1)In the age of ever-changing social health care needs, reliance on the knowledge acquired during student life is insufficient to meet the clinical needs, and nursing staff are required to strengthen their self-directed learning ability to adopt to the fast-expanding nursing knowledge.(2) Self-directed learning ability is the foundation of the lifelong learning for nursing students.(3)To make nursing students equipped with the ability to learn throughout their nursing career, Self-directed learning can be an effective and innovative approach which has shown its efficacy to promote critical thinking and professional development.(4,5)Study have shown that self-directed learners constantly organize, monitor and evaluate their study plans and achieve better academic result than non-self-directed learners.(6)

Historically, self-directed learning (SDL) was introduced by Knowles (1975) as a process in which learners show self-initiation in diagnosing their learning needs, establish their learning goals, identifies learning resources, select and use appropriate learning strategies and evaluate learning outcomes with or without support from others.(4) Self-directed learning emphasizes the active role of learner as it allows them to have control and regulate their academic learning. It revolves around the notion of creating a desire to learn something new and qualify oneself for more advanced abilities in the students' area of interest.(5)Therefore, there is growing recognition of the need to incorporate SDL in nursing education and improve students' SDL ability by changing their learning environment to include new student-centred innovative teaching learning methods.

Self-directed learning can be planned and implemented according to the characteristic of the learner and learning environment. It is categorised as facilitated learning where the teachers provide learning content through discussion, mail or online forums, and self-paced learning where learners show motivation and initiation towards learning and are competent to choose a suitable course and gather learning materials.(7)

Worldwide, the higher education system is gradually shifting from teacher centred to student centred where students are considered as active learners and they are responsible to redirect their learning to specific competencies, foster lifelong learning and gain professional development. In context of health professionals, SDL is an approach to remain flexible, proficient and resourceful to cope of with ever-changing healthcare need of society and medical knowledge.(8,9)

Nurses' competency level has been associated with patients' outcome in health care.(10) Therefore, by being a self-directed learner, a nursing student can not only achieve academic success but also foster a habit to remain up-to-date with the health care modalities and provide safe care to patients.(10,11)Self-directed learning permit to enlarge their theory base, enabling nurse’s professional development which is crucial for the quality of nursing care.(12)

Self-directed learning is seen as a prerequisite to meet professional development needed for working at complex and ever changing specialized clinical environment.(9) The rising demands of medical technology and evidence based clinical decision making has added the requirement for professional development through Self-directed learning.

Self-regulation and autonomy in learning both require motivation. Self-determination theory states that a learner will behave independently when they either like the activity or feel it is necessary to achieve a significant or valuable goal.(13)Since motivation is an essential mediator for both engagement and self-directed learning, strategies which has shown its significance on promoting students’ engagement and creating fascinating learning environment for favourable outcome needs to be considered.

With the advancement and accessibility of new technologies, a wide range of innovative teaching approach has been developed to facilitate SDL in the field of health profession education. In the current era of e-learning and SDL, game-based learning is growing as a more attractive educational approach that integrate gaming elements with learning materials and boosts students’ engagement, encourage active learning and increases learner’s achievement compared to conventional learning method.(14)In recent years, there has been a growing body of evidence that demonstrates GBL can enhance student engagement, boost their motivation to learn, and improve learning outcomes.(15–18)

Game based learning (GBL) typically referred to as “serious game”, “educational game” and “gamification” has the potential to bring immersive experience for nursing students and

improve their study outcomes.(14) As opposed to more traditional instructional approaches, GBL has the ability to render learners with opportunities of active learning, solving clinical problems and gaining clinical experiences in a risk-free environment. (19–21)

Game based learning allows students to make mistakes and learn from them without endangering real patients. This builds confidence and competences before entering clinical practice. It inherently provides a level of engagement and motivation that traditional teaching methods may struggle to achieve. Nursing students can be more invested in their learning when presented with challenging and interactive game scenarios. Since game can produce instant feedback on student's actions and decisions, helping them understand the consequences of their choices. This promotes self-assessment and continuous improvement. Additionally, the immersive and interactive nature of game can lead to improved information retention. (21–23)Therefore game-based learning can be used as a self-directed learning strategy for ongoing professional development and continuing education to keep nurses up to date with the latest practices and guidelines. Stakeholders in nursing education should be encouraged to promote and support the integration of self-directed learning with innovative approaches into nursing curricula to enhance the quality of nursing education and improve patient care outcomes.

The proposed framework for self-directed learning in this study is developed by mapping the psychological constructs of self-determination theory (SDT) and elements of gaming into the paradigm that the autonomous motivation for initiation of self-directed learning and favorable learning outcome can be fostered by satisfying the psychological needs of autonomy, competence, and relatedness through gamification. Gamification is a transformative approach to education, combining games and learning to engage the current generation of young and technology-savvy learners. By integrating gaming elements with self-determination theory and Garrison's self-directed learning model, educators can create a more engaging, motivating, and effective learning environment that encourages learners to take charge of their educational journey and promote self-directed learning.

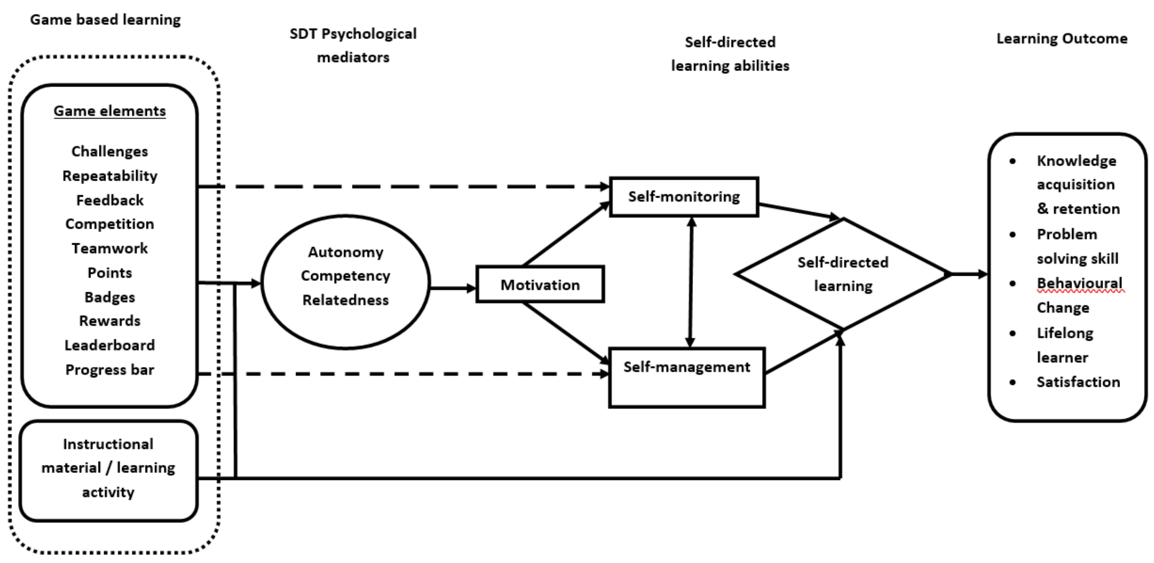

Figure 1. Conceptual framework for Self-directed learning adopted from Landers' theory of gamified learning (2014), Self-determinant theory and Garrison's model of self-directed learning

Figure 1 illustrates this framework. Gaming elements can satisfy the psychological needs of SDT by providing learners with a sense of control, and opportunities to develop mastery and social connections through collaborative and competitive activities. The framework proposes that if participating in a learning activity integrated with gaming elements provides the learner with a sense of autonomy and competence as well as a sense of belonging, then motivation would be enhanced and learners are more likely to engage deeply and persistently with their learning tasks resulting in knowledge retention, problem-solving skill and improvement in overall academic performance.

LITERATURE REVIEW

There is a growing recognition of the need to incorporate self-directed learning in nursing education to promote critical thinking, lifelong learning and professional development among nursing students.(4,5,7,24)Self-directed learning requires students to take responsibility for their learning process, set goals, and identify resources for acquiring knowledge.(11,25) While some nursing students may excel in self-directed learning, others might face challenges in this approach due to various factors such as prior educational experiences, learning preferences, and confidence levels.(11)A systematic review and meta-analysis by Nazarianpirdosti M, revealed that the level of SDL in nursing students is far from the desired level and future studies are suggested to evaluate the effect of various self-directed learning strategies and impact of self-directed learning on nursing students' learning outcomes and factors facilitating and inhibiting SDL.(26)Similarly, SDL ability of nursing undergraduates were found low in a cross-sectional study using canonical correlation analysis by Li-Qing Tang et.al. Students' perception of learning (SPL) and academic self-perceptions (SASP) were positively correlated with self-management ability and cooperative learning ability. Study recommended that nursing educators can improve

students' SDL ability by changing their learning environment, using, for example, new student-centred teaching methods.(2)Therefore, it is crucial to explore the various support mechanisms like integration of modern technology that can be implemented to foster self-directed learning effectively.

A descriptive study done among Saudi nursing and emergency medical services undergraduates revealed that students' perceptions of their learning environment played a significant role in determining their level of self-directed learning readiness and academic performance. Study recommended to deliver a supportive learning environment considering good teaching, clear goals and standards, appropriate assessment, appropriate workload, and giving importance on independence, encouraging students to engage in the process of self-directed learning which can, consecutively, enhance their academic performance.(6)

Research have shown a positive correlation between self-directed learning, and academic achievement. In student-centred learning environment, learners with higher self-regulated learning ability have shown better academic performance than non-self-regulated learners. This depicts the need of self-directed learning strategies in learner-oriented learning environment. One study has suggested that innovative educational methods along with SDL can be more beneficial, as learners can control their own speed and learning process.(2)

Literatures have revealed various elements of self-directed learning, like self-regulation, motivation, personal responsibility and autonomy. (11)Several studies have been conducted in different countries to describe nursing students' level of SDL abilities and have shown conflicting results. In this regard, the level of SDL in nursing student was reported to be high in European countries, Thailand, Saudi Arabia and Turkey, whereas moderate in Iran and low in Australia.(10) A systematic review and meta-analysis by Nazarianpirdosti Met al. Identified moderate level of SDL in nursing students, which does not seem to be sufficient.(26) Considering the importance of SDL in nursing education, it is essential to introduce instructional approaches for promoting SDL in nursing education.

A pilot randomized controlled trial done in Korea to evaluate the effectiveness of self-regulated learning based on augmented reality technology as an innovative learning method for nursing students revealed that self-regulated learning competency were significantly improved after the intervention and to maintain the SRL competency, learners need to seek information independently, organize and transform it and seek social assistance from peers and teachers. Therefore, students should be provided with suitable information and assistance for successful self-directed learning.(27)A systematic review by Murad Mand colleague also identified that the benefit of SDL increases when learner are involved in selecting their learning resources and strategies. (8)

At present, game-based learning is a new trend and is being extensively applied in health profession education to bring immersive experience for student and improve their study outcomes. As opposed to more traditional instructional approaches, GBL has shown its

effectiveness in encouraging engagement and raising the enthusiasm in students.(14)A comparative pilot study by Grech J has highlighted the significance of gamification in increasing student engagement and interaction.(28) Castro MJ et al. pointed that implementation of educational games which consider response time and correct answer favours competitiveness and motivates students to actively participate in their learning process.(29)

A Norwegian randomized controlled trial aimed to examine the effectiveness of game-based learning compared to traditional learning concluded that though there was no significant improvement in examination result, frequent use of game appeared to influence the examination outcome positively.(30)Identical result was shown by the randomized controlled trial from France by Blanie A where one hundred forty-six nursing students were assessed for the impact of simulation by gaming and found greater level of satisfaction and motivation among experimental group then control group though there was no significant educational difference, neither immediate nor one month later. (31)But a similar trial by Chang YS et.al. suggested that game-based learning could enhance nursing student's retention of knowledge.(32)

Over the recent years, mounting evidence suggests that game-based learning has been more efficient than traditional teaching methods as it provides an interactive and engaging environment that promotes intrinsic motivation, autonomy, immediate feedback, progress tracking, scaffolded challenges, and personalization which facilitate self-directed learning by empowering learners to take control of their learning, make decisions, and actively engage in the learning process. Additionally, it has been demonstrated to support student relationships by fostering teamwork, reciprocity, and cooperation. (14–17,21,29,30,33–39)These benefits of game-based learning seem to enhance the self-directed learning ability and could be a possible approach to nursing education. However, this approach has rarely been examined for nursing education. (40)This fact demands research focused on exploring the widespread use and impact of GBL on improving self-directed learning and learner centred nursing undergraduate curriculum.

Research is needed to identify the best practices for seamless integration, ensuring that games complement other instructional methods and align with the nursing program's objectives. Although some studies have shown the benefits of game-based learning in nursing education, more research is needed to understand how game-based learning compares with conventional approaches in terms of knowledge acquisition, skill development, and overall student satisfaction. While there is evidence that game-based learning can enhance short-term engagement and knowledge retention, there is a lack of comprehensive studies that investigate the long-term impact of these educational games on nursing students' academic outcomes. A systematic review to explore student' experience and learning when using game-based learning and to understand its uses in the nursing curriculum by Tavares N, concluded that a game-based learning is an important alternative

to traditional teaching methods and can produce a positive short-term impact on learning and knowledge retention, whilst the long-term impact of GBL on knowledge retention, learning and clinical skills generated mixed results.(16)Therefore, a research that follows students beyond their immediate exposure to game-based learning and assesses their knowledge retention would be valuable.

As game-based learning has been reported to bring positive change in behaviour and learning with high satisfaction among learner, it seems to be a promising tool to improve learning outcomes by strengthening learning behaviour and attitude towards learning. (29)However, the current research finding is too limited to provide educators with evidence-based recommendation on when and how specific gaming elements should be used to enhance the learning behaviour. (20)So, it's essential to assess whether game-based learning is equally effective for all nursing students to enhance their self-directed learning ability. Understanding the suitability and potential challenges for diverse learners can help tailor educational approaches accordingly.

RESEARCH GAP ANALYSIS

SN	Title, Author, Year	Study Objectives, Design and Sample size	Findings of the study (outcome) and conclusions by Author	Remarks by scholar
1	The effectiveness of self-directed learning in health professions education: a systematic review.<br><br>Murad, M.H., Coto-Yglesias, F., Varkey, P., Prokop, L.J. and Murad, A.L. (2010)(8)	To determine the effectiveness of SDL in improving learning outcomes in health professionals.<br><br>59 studies<br><br>8011 samples	Compared with traditional learning methods, SDL was moderately more effective in the knowledge domain and likely to be as effective in the domains of skills and attitudes.	• Out of 59, only 6 studies were among nurses<br>• All nursing studies were non randomized, pre/post design, unblinded outcome<br>• None of the studies used game-based learning as SDL strategy<br>• No RCT<br>• No Indian

				study
2	Evaluation of Self-Directed Learning in Nursing Students: A Systematic Review and Meta-Analysis<br>Mohammad Nazarianpirdostiet.al.(26)<br>2021	to analyze the existing research on the level of self-directed learning in nursing students<br>12 articles, 3830 samples	SDL in nursing students is far from the desired level, future studies are suggested to evaluate the effect of various SDL strategies and its impact on students' learning outcome	• None of the study from India<br>• Self-directed learning ability of nursing needs to be assessed and improved
3	Self-Regulated learning strategies for nursing students: A pilot randomized controlled trial<br>An J, Oh J, Park K(27)<br>2022	To compare the effect of the use of augmented reality as an innovative learning method and the use of a textbook as a conventional learning method.<br>Pilot RCT<br>62 sample size	• Knowledge score, perceived learning and learning flow improved significantly after the intervention in both group.<br>• Study concluded that the use of innovative educational technology was not a superior method to improve SRL	It is necessary to conduct larger RCT to test effectiveness of self-directed learning using efficient innovative educational technology for a longer period of time.

			competency for nursing students.	
4	The use and impact of game-based learning on the learning experience and knowledge retention of nursing undergraduate students: A systematic literature review<br><br>Nuno Tavares, 2022(16)	To explore student experience and learning when using game-based learning and to understand its uses in the nursing curriculum, Systematic literature review, 17 papers from 4 different countries	• High level of student experience, engagement and motivation<br>• Increased level of anxiety in time limited teaching activities<br>• Gender difference were found<br>• Positive short term impact on knowledge retention	• No Studies from Asian countries<br>• Research exploring indepth impact of GBL on nursing students<br>• More studies are required for analysis of long term retention<br>• Students' perception on GBL activities should be considered.
5	Comparative value of a simulation by gaming and a traditional teaching method to improve clinical reasoning skills necessary to detect patient deterioration: a randomized study in nursing student.	To compare the respective educational value of simulation by gaming and traditional teaching method to improve	• No significant educational difference, neither immediate nor 1 month later was observed between experimental and control group. However satisfaction	• Additional studies are necessary to seek the efficacy of game based learning.

	Blanie et al. 2020(31)	clinical reasoning skills<br>RCT<br>146 samples (73 in each group)	and motivation were found to be greater with the use of simulation by game.	
6	Digital game-based learning: a supplement for medication calculation drills in nursing education.<br><br>Foss B, Lokken A, et.al., 2014(30)	To examine whether baccalaureate student nurse who played the medication game as a supplement to lectures and task-solving improve their examination result compared to a control group who used lectures and task-solving only.<br>RCT, 201 samples	• No significant difference between groups in examination pass rate.<br>• Medication game did not significantly improve examination results, but that using the game frequently appeared to influence the examination outcome positively.	• SDL ability is not tested.<br>• Longer gaming period should be studied to see how time affects the influence of game-based learning on knowledge retention and examination output.

NATURE OF THE KNOWLEDGE GAP IDENTIFIED

Research Gap Types	Type of research gap used
Knowledge Gap	• No evidence of evaluation of self-directed learning among nursing students in Indian setting.

Methodological Gap	•	No RCT has been identified for evaluating impact of SDL with game-based learning
Practical-knowledge Gap	•	There is practical knowledge gap in long term impact of game-based learning on undergraduate nursing curriculum
Population Gap	•	No studies for evaluating effectiveness of SDL and game-based learning among nursing students in India

RESEARCH QUESTIONS

Does the use of game-based learning as an innovative self-directed learning strategy improve nursing undergraduates' self-directed learning abilities in comparison to conventional learning method?

HYPOTHESIS

H_1: The incorporation of game-based learning as an innovative self-directed learning strategy will be superior to conventional learning method in improving self-directed learning abilities among nursing undergraduates.

H_2: Nursing students in game-based learning group will have higher knowledge scores than those in conventional learning group.

AIM

To compare the effect of the game-based learning as an innovative self-directed learning strategy with conventional self-directed learning strategy on knowledge acquisition, and self-directed learning abilities in nursing undergraduates through a randomized controlled trial.

OBJECTIVES

1. To assess the effect of game-based learning as an innovative self-directed learning strategy on knowledge acquisition among nursing undergraduates.
2. To assess the effect of conventional learning method on knowledge acquisition among nursing undergraduates.
3. To evaluate the effect of game-based learning as an innovative self-directed learning strategy on self-directed learning abilities among nursing undergraduates.

4. To evaluate the effect of conventional learning method on self-directed learning abilities among nursing undergraduates.
5. To compare the impact of game-based learning and conventional learning method on knowledge acquisition among nursing undergraduates after 12 weeks of intervention.
6. To determine whether game-based learning is superior to conventional learning in improving self-directed learning abilities in nursing undergraduates after 12 weeks of intervention.

METHODS

STUDY DESIGN

It is a prospective, randomized, controlled, data analyst-blinded, single centric superiority study with two parallel groups allocated at a 1:1 ratio and follow-up period of 12 weeks to identify the impact of self-directed learning strategy using educational games as an innovative learning method for nursing students in the experimental group compared with the conventional learning group as control.

PARTICIPANTS, INTERVENTIONS, AND OUTCOMES

STUDY SETTING

This trial will be conducted at Smt. Radhikabai Meghe Memorial College of Nursing (SRMMCON), a renowned nursing college located in Sawangi (Meghe), Wardha, Maharashtra, India. SRMMCON is affiliated with the Datta Meghe Institute of Higher Education & Research (Deemed to be University) and is recognized by the Indian Nursing Council (INC) and the Maharashtra Nursing Council (MNC). It is dedicated to providing high-quality nursing education and training and offers undergraduate, postgraduate, postgraduate diplomas and doctoral program in nursing.

ELIGIBILITY CRITERIA

Inclusion criteria:

1. Participants must have been enrolled in an accredited undergraduate nursing program (B.Sc. Nursing).
2. Participants in their first or second year of study, who are most likely equipped with basic nursing knowledge but are still improving their clinical skills and self-directed learning (SDL) routines.

3. Participants who give informed consent and show a willingness to take part in the study.
4. Participants should have basic proficiency in English language, as SDL materials and assessments is in English language.
5. Participants must have access to a computer or laptop or tablet or smart phone, and internet connectivity, as SDL strategies rely on online resources and e-learning platforms.

Exclusion criteria:

1. Students who have received previous classes or training in antimicrobial resistance, antimicrobial stewardship and infection control should be excluded to prevent any potential influence on the intervention's outcomes.
2. Students who have a record of irregular attendance in class, as SDL requires dedication and regularity.
3. Students who continuously fail to engage in intervention or express a desire to withdraw can be removed from the study.

Sample size

The study hypothesize that the self-directed learning abilities and knowledge acquisition among participants attending the game based learning would be superior to those attending the conventional learning. The sample size is determined using G*Power (Version 3.1.9.7). Assuming a significance level of 0.05 and effect size of 0.5 (based on meta-analysis study* which estimated a medium effect size in favor of game-based learning over learning without gamification), 64 participants per arm will be required to achieve a statistical power of 80%. Considering a dropout rate of 10%, 70 participants per arm, 140 participants in total, is required.

*Bai, S., Hew, K.F., & Huang, B. Does gamification improve student learning outcome? Evidence from a meta-analysis and synthesis of qualitative data in educational contexts. *Educ Res Rev.2020;1(30):100322.*

- Census sampling will be done to eliminate the selection bias. All the basic Bsc.Nursing students from two semester will be enrolled in study with hundred in each experimental and control group. Total 200 students is planned to be enrolled.

Recruitment

The accessible population for this study is undergraduate nursing students at Smt. Radhikabai Meghe Memorial College of Nursing (SRMMCON), specifically first, second, and third semester students. The total accessible population consists of 200 students, with 100 students in each first and third semester. To ensure equal representation, stratified random

sampling will be used. The population will be divided into strata based on semester and 70 students will be randomly selected from each stratum. Each eligible student will be assigned a unique identification number. The study will select 70 students from the first semester students' list and another 70 from the third semester students' list using a random number generator.

Assignment of interventions (for controlled trials)

Allocation

Sequence generation

Researcher will use computer-generated random numbers to allocate participants to one of the two groups. The randomization method employs block randomization with block size of 4 and 8. Stratification is based on the semester of their study, as this factor may influence the study outcome. Independent personnel not involved in the study will utilize random sequence generator software to generate a random sequence, randomly allocating subjects in a 1:1 ratio and dividing them into two groups.

Allocation concealment mechanism

Random numbers will be placed in coded, opaque envelops and entrusted to personnel not involved in study until the random allocation and intervention is initiated.

Blinding (masking)

An independent assessor, not involved in intervention delivery will conduct outcome assessment for an educational intervention. Due to the nature of the intervention, blinding the researcher is not possible. The data analyst will be blinded in the study. The data will be coded with neutral labels, ensuring unbiased analysis without revealing group assignments.

Interventions

Researcher will provide detailed information about purpose and procedures of the study to the participants. They will be ensured for the anonymity of participants' personal information and collected data. Participants will be made aware of approximate time needed to complete the questionnaire (around 15-25mins). They will be informed that there will be no penalty for not participating and they can withdraw at any time during the study. They will be assured that their responses would not have any effect on their academic ground and it will be kept confidential with researcher. A written informed consent will be taken from the subjects willing to participate in the study.

After signing an informed consent form, participants will undertake a pretest covering knowledge, self-directed learning abilities, and perceived learning competency. Following that, two batches of students on the basis of their academic semester will participate in a 90-minute structured teaching session on self-directed learning, where the students will be

engaged in group activity and the concept of self-directed learning, its importance and process will be discussed with them.

Self-directed learning module:

S. N	Activity	Duration	T/L Method & Media
**1**	Formulation of group	15min	
**2**	• A common learning goal will be established<br>• Each group will be given a topic to carry out SDL to achieve common goal<br>• Each group will discuss the resources required for the topic, the methods of SDL they will be using to achieve the common goal<br>• Each group will present their SDL plan	30min	Chart paper for the presentation
3	From the group activity- the concept of SDL, its importance, process will be discussed with the students.<br>• Establish learning goals<br>• Locate & access resources<br>• Adopt & execute learning activities<br>• Monitor & evaluate performance<br>• Reassess learning strategies	45 min	PPT and interactive discussion with examples

Subsequently, the participants will be randomly assigned to either the intervention or control groups, using numbered envelop, by simple random sampling determined by a randomization list.

<u>In Experimental group</u>

After a session on Self-directed learning, experimental group will be further divided into small teams and then exposed to AMS game. Researcher will act just as a facilitator and share gaming screen with the team and encourage group discussion.

Antimicrobial stewardship (AMS) game is developed by the Commonwealth Partnerships for Antimicrobial Stewardship Programme, (CwPAMS*), led by Commonwealth Pharmacists Association (CPA) and Tropical Health Education Trust (THET) in partnership with Focus Games Ltd. AMS game is designed to teach the basics of Anti-microbial resistance, Anti-microbial stewardship and infection control, and also how they intertwine to impact global health. Players are challenged and entertained in this team game as they tackle various

scenarios, test their knowledge, and change behaviours that can ultimately save lives of people.
Immediate postintervention test following gaming session will be taken and schedule for next online AMS gaming session on first weekend along with zoom login ID and password will be shared to the experimental group participants. And login details for consecutive AMS gaming will be shared to them a day before weekend for two following weeks in their email. Altogether four gaming session will be conducted in a period of four weeks.

In Control group

After Self-directed learning session, learning material on antimicrobial resistance, antimicrobial stewardship and infection control will be shared to all the participants and one hour will be given to them for self-study. Immediate post-test will be taken after one hour of self-study and encourage to do self-learning with provided learning material for four weeks.

Strategies to improve adherence to interventions

To improve adherence to intervention protocol, text message in WhatsApp and email will be send to all the participants. The researcher will interact with participants weekly for one month on a Zoom online platform to provide guidance on self-directed learning and answer questions about learning methods. However, no lectures will be given on the learning content so that outcome variables are not affected.

To promote participant retention and minimize the risk of communication between participants in the control and experimental groups during the 4-week of follow-up period, we will implement several strategies. Participants will be instructed on maintaining confidentiality of group assignments and intervention details. The control group will be assured of getting access to the AMS game at the end of the study to reduce tendency for seeking premature information. Only experimental group will get login code for zoom platform for playing AMS game, ensuring only the assigned group can attend each session. During the game-based learning session, all participants will be asked to keep their cameras on to ensure that only the experimental group participants are present.

Strategies for promoting participant retention and ensuring complete follow-up

The researcher will follow up with participants who miss sessions or show non-compliance, offering support and solutions if needed via email, WhatsApp or phone call. Participants with workload, technological issues, or personal difficulties will be offered solutions (e.g., flexibility in gaming session or SDL discussion session, technical support). Participants who deviate but do not withdraw will be included in an intention-to-treat analysis to preserve randomization integrity. Those who fail to engage or express a desire to withdraw will be removed from the study and documented with reason of withdrawal for proper interpretation of study result and impact on sample size. If attrition is high, additional participants will be randomized to fill gaps and maintain the study's statistical power.

Outcomes

The outcome measurements will be conducted at baseline, immediately postintervention, 4 weeks postintervention, and 12 weeks postintervention using the same data collection tool.

Primary Outcome:

1. Self-Directed Learning abilities Improvement:

The primary outcome would be improvement in self-directed learning (SDL) abilities among nursing undergraduates after intervention. This will be measured by using validated SDLI tool, which assess key SDL competencies such as:

a. Learning motivation
b. Planning and implementing
c. Self-monitoring and
d. Interpersonal communication

The difference in Pre- and Post- intervention SDLI scores between the experimental and control groups would highlight the effectiveness of game-based learning in improving SDL abilities.

2. Knowledge Acquisition

Another primary outcome is improvement in knowledge acquisition in the topic antimicrobial resistance, antimicrobial stewardship & infection control. This will be assessed by using structured questionnaire developed and validated by researcher. The difference in scores between the game-based learning and conventional SDL group would highlight the effectiveness of each strategy in improving knowledge acquisition and retention.

Secondary Outcome:

1. Cognitive, Affective and Psychomotor perceived learning Improvement:

Improvement in the cognitive, affective and psychomotor domain of CAP perceived learning competency scale on postintervention score of experimental group will signify positive impact of intervention.

Participant timeline

A schematic diagram presenting enrolment, random allocation, intervention, follow-up and data analysis of the study is shown below;

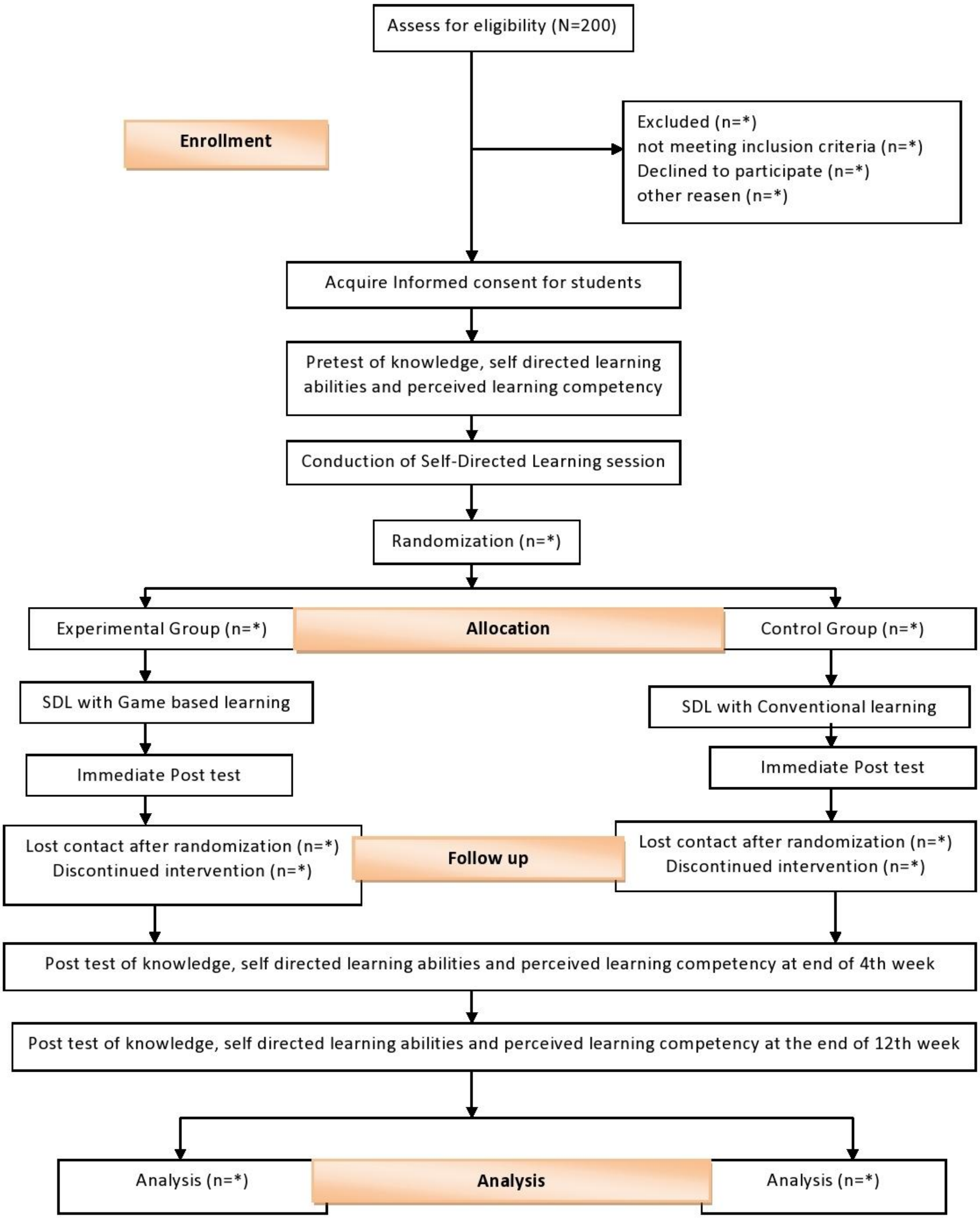

Figure 2. Schematic diagram for study

Data collection, management, and analysis

Data collection methods

The researcher will use a structured and pre-tested data collection inventory to gather study data. The data will be entered into the study database promptly. After obtaining written consent, a self-administered inventory will be distributed and collected from students. Data collection will occur at four time points: preintervention, immediately postintervention, 4 weeks postintervention, and 12 weeks postintervention using the same inventory.
Data collection inventory comprises of four sections;

A. **Socio-demographic information:** age, gender, semester, place of residence and previous exposure to game-based learning.

B. **Knowledge questionnaire:** A 15-item multiple-choice questionnaire on antimicrobial resistance, antimicrobial stewardship, and infection control will be used to assess knowledge. The score ranges from 0-15, with scores categorized as adequate knowledge (above 75%), moderately adequate knowledge (between 51-75%), and inadequate knowledge (less than 50%). Each item will be tested for validity and reliability before the study.
Expert evaluation will confirm content validity, with a panel of subject matter experts assessing relevance, clarity, and alignment with study objectives. Cronbach's alpha will be used to determine internal consistency, with a Cronbach's alpha value of 0.70 or higher indicating acceptable reliability. Test-retest reliability will be assessed by administering the questionnaire to a sample of participants at two different time points (with an interval of two weeks). Intraclass correlation coefficients (ICC) will be used to assess results' consistency over time, with an ICC value greater than 0.75 indicating strong reliability.

C. **Self-Directed Learning Instrument (SDLI)**: The Self-Directed Learning Instrument (SDLI) is a reliable tool developed by Cheng et al. (2010) with good internal consistency (Cronbach's total scale was α = 0.916). Cadorin L et al. (2017) recommend SDLI for assessing SDL abilities among nursing students and nurses due to its excellent methodology quality, Cronbach α ranging from 0.73 to 0.91, and structural validities. Content validity was supported by two rounds of Delphi study. It is comprised of 20 items categorized into four domains:
    a. Learning motivation: six items (α = 0.80),
    b. Planning and implementing: six items (α = 0.86),
    c. Self-monitoring: four items (α = 0.78), and
    d. Interpersonal communication: four items (α = 0.76).

The Self-directed learning instrument (SDLI) metric uses a five-point Likert scale to measure self-assessment of self-directed learning (SDL) abilities. Scores range from 1 to

5, with "strongly disagree" indicating very low level of (self-assessed) abilities, and "strongly agree" indicating high level of (self-assessed) SDL abilities. All the statements are stated positively and the total possible score ranges from 20 to 100, with higher scores indicating higher SDL abilities.

D. **Cognitive, Affective & Psychomotor (CAP) perceived learning scale**: The study will utilize the CAP Perceived Learning Scale, a nine-item, 6-point Likert scale, developed by Rovai et al. (2009), to measure perceived cognitive, affective, and psychomotor learning in game-based and conventional learning environments. The scale is a reliable and valid tool with internal consistency of 0.79 and supported concurrent validity. It generates an overall score representing perceived learning across three domains: cognitive learning, affective learning, and psychomotor learning.

Subscale cognitive learning describes a students' ability to recall or recognition of knowledge, affective learning is the positive attitude towards the subject matter, and psychomotor learning is the capacity to perform tasks (Rovai et al., 2009). In CAP Perceived Learning Scale, statements 1,2 and 5 measures perceived cognitive learning; statement 4,6, and 9 measures perceived affective learning and statement 3, 7, and 8 measures perceived psychomotor learning. Statement two and seven are inversely scored while the other statements are scored in a standard pattern. Total scores can range from a maximum of 54 to a minimum of 0 and CAP subscale scores can range from maximum of 18 to minimum of 0. A high CAP Perceived Learning Scale score is an indicator of a keen perception of learning by students participating in the study (Rovai et al., 2009).

Self-directed learning instrument (SDLI) and CAP Perceived learning Scale will be used for the study in its original form without any modification. Participant information with personal identifiers will be stored in paper-based form in a locked filing cabinet in a secure and locked private space. To ensure accuracy and completeness, study data will be reviewed by the researcher.

Data Management & Monitoring

The study will use SPSS software for data management, including entry, validation, coding, cleaning, and preparing the final database. The researcher will maintain and store study records, including informed consent. The Data Monitoring Committee (DMC) is not essential due to nature, scale, and resources availability. Alternative monitoring measures will be implemented to ensure scientific and ethical integrity. Data management will comply with institutional ethics committee for participant protection and confidentiality. Regular review of trial progress will ensure data quality and accuracy.

Statistical Methods

The study will use SPSS version 21, with tests like Shapiro-wilk, skewness, and kurtosis to evaluate the normal distribution of variables. Pre-test homogeneity will be assessed using descriptive statistics, independent t-tests, chi-square tests, and Fisher's exact test. ANOVA will compare differences in SDL ability, PL competency, and knowledge between groups. Regression analysis will explore the relationship between independent variables and dependent outcomes. Differences between pre-test and post-test will be analyzed using paired t-test. All statistical analysis will be two-sided, with a p-value less than 0.05 considered statistically significant.

Translatory Component (Conceptualized)

- The research could provide insights into the effectiveness of game-based learning as a self-directed learning approach for nursing students. It will demonstrate how game-based learning improves self-directed learning abilities of nursing students.

- It could shed light on whether incorporating game elements in teaching learning, improve learners' engagement and long-term learning outcome.

Ethics and Dissemination

IEC Approval

The study will be conducted in accordance with the Declaration of Helsinki 2008. Scholar will acquire ethical clearance from institutional ethical committee of DattaMegha Institute of Higher Education and Research with the help of PhD cell. Any amendments to the protocol and procedure will be done only after receiving approval from the IEC of DMIHER.

Consent

Researcher will provide detailed information about purpose and procedures of the study to the participants. They will be ensured for the anonymity of participants' personal information and collected data. Participants will be made aware of approximate time needed to complete the questionnaire (around 15-25mins). They will be informed that there will be no penalty for not participating and they can withdraw at any time during the study. They will be assured that their responses would not have any effect on their academic ground and it will be kept confidential with researcher. A written informed consent will be taken from the subjects willing to participate in the study.

Confidentiality

To safeguard confidentiality, a unique alpha-numeric identifier will be assigned to each participant to anonymize personal information and data.

Dissemination plan

Scholar plan to disseminate study results in peer-reviewed scientific journals and academic conferences, targeting educators and practitioners, students, academic researchers, educational organizations and policy makers, thereby fostering the widespread sharing of scientific knowledge and the advancement of best practices.

Funding

None.

Competing interests

No competing interests. The AMS gaming application for study will be purchased retail; Focus Game has no role in the study.

Access to data

Primarily, access to the data will be restricted to researcher and data analysts. Later the study protocol and data analysis after completion of study will be available from the primary researcher upon request.

Posttrial care

To address beneficence and justice to the participants, control group will get access to AMS gaming and experimental group will get learning material after 12th week follow up post-test.

References

1. Institute of Medicine (US) Committee on the Robert Wood Johnson Foundation Initiative on the Future of Nursing at the Institute of Medicine. The Future of Nursing: Leading Change, Advancing Health. Washington (DC): National Academies Press (US); 2011.

2. Tang LQ, Zhu LJ, Wen LY, Wang An-Shi and Jin YL, Chang WW. Association of learning environment and self-directed learning ability among nursing undergraduates: a cross-sectional study using canonical correlation analysis. BMJ Open. 2022 Aug;12(8):e058224.

3. Al Moteri MO. Self-Directed and Lifelong Learning: A Framework for Improving Nursing Students' Learning Skills in the Clinical Context. Int J Nurs Educ Scholarsh. 2019 Nov;16(1).

4. Greveson GC, Spencer JA. Self-directed learning–the importance of concepts and contexts. Med Educ. 2005 Apr;39(4):348–9.

5. Mamary E, Charles P. Promoting self-directed learning for continuing medical education. Med Teach. 2003 Mar;25(2):188–90.

6. Alotaibi KN. The learning environment as a mediating variable between self-directed learning readiness and academic performance of a sample of saudi nursing and medical emergency students. Nurse Educ Today. 2015 Nov;36:249–54.

7. Robinson JD, Persky AM. Developing Self-Directed Learners. Am J Pharm Educ. 2020 Mar;84(3):847512.

8. Murad MH, Coto-Yglesias F, Varkey P, Prokop LJ, Murad AL. The effectiveness of self-directed learning in health professions education: A systematic review. Vol. 44, Medical Education. 2010. p. 1057–68.

9. Cadorin L, Bressan V, Palese A. Instruments evaluating the self-directed learning abilities among nursing students and nurses: a systematic review of psychometric properties. BMC Med Educ. 2017 Nov;17(1):229.

10. Visiers-Jiménez L, Palese A, Brugnolli Anna and Cadorin L, Salminen L, Leino-Kilpi Helena and Löyttyniemi E, Nemcová J, et al. Nursing students' self-directed learning abilities and related factors at graduation: A multi-country cross-sectional study. Nurs Open. 2022 Feb;9(3):1688–99.

11. Wong FMF, Tang ACY, Cheng WLS. Factors associated with self-directed learning among undergraduate nursing students: A systematic review. Nurse Educ Today. 2021 Jun;104:104998.

12. Shen WQ, Chen HL, Hu Y. The validity and reliability of the self-directed learning instrument (SDLI) in mainland Chinese nursing students. BMC Med Educ. 2014 May;14:108.

13. Ryan RM, Deci EL. Intrinsic and Extrinsic Motivations: Classic Definitions and New Directions. Contemp Educ Psychol. 2000 Jan;25(1):54–67.

14. Xu M, Luo Y, Zhang Y, Xia R, Qian H, Zou X. Game-based learning in medical education. Front Public Health. 2023 Mar;11:1113682.

15. Yaqi X, Lau Y, Cheng LJ, Lau ST. Learning experiences of game-based educational intervention in nursing students: A systematic mixed-studies review. Vol. 107, Nurse Education Today. Churchill Livingstone; 2021.

16. Tavares N. The use and impact of game-based learning on the learning experience and knowledge retention of nursing undergraduate students: A systematic literature review. Vol. 117, Nurse education today. NLM (Medline); 2022. p. 105484.

17. Kuruca Ozdemir E, Dinc L. Game-based learning in undergraduate nursing education: A systematic review of mixed-method studies. Vol. 62, Nurse Education in Practice. Elsevier Ltd; 2022.

18. Gallegos C, Tesar AJ, Connor K, Martz K. The use of a game-based learning platform to engage nursing students: A descriptive, qualitative study. Nurse Educ Pract. 2017 Nov 1;27:101–6.

19. van Gaalen AEJ, Brouwer J, Schönrock-Adema J, Bouwkamp-Timmer T, Jaarsma ADC, Georgiadis JR. Gamification of health professions education: a systematic review. Vol. 26, Advances in Health Sciences Education. Springer Science and Business Media B.V.; 2021. p. 683–711.

20. Gentry SV, Gauthier A, Ehrstrom BLE, Wortley D, Lilienthal A, Car LT, et al. Serious gaming and gamification education in health professions: systematic review. Vol. 21, Journal of Medical Internet Research. JMIR Publications Inc.; 2019.

21. Akl EA, Sackett KM, Erdley WS, Mustafa RA, Fiander M, Gabriel C, et al. Educational games for health professionals. Cochrane Database Syst Rev. 2013 Jan;(1):CD006411.

22. M MS, C K N, Srivastava TK. Digital Game-Based Learning (DGBL) Through Kahoot: A Learner-Centric Approach in Nursing Education - Systematic Review. International Journal of Science and Healthcare Research. 2022 Jul 20;7(3):14–9.

23. Sannathimmappa MB, Nambiar V, Aravindakshan R. Learning out of the box: Fostering intellectual curiosity and learning skills among the medical students through gamification. J Educ Health Promot. 2022 Mar;11:79.

24. Tekkol IA, Demirel M. An investigation of self-directed learning skills of undergraduate students. Front Psychol. 2018 Nov 23;9(NOV).

25. Patra S, Khan A, Upadhyay M, Sharma R, Rajoura O, Bhasin S. Module to facilitate self-directed learning among medical undergraduates: Development and implementation. J Educ Health Promot. 2020 Sep 1;9(1).

26. Nazarianpirdosti M, Janatolmakan M, Andayeshgar B, Khatony A. Evaluation of Self-Directed Learning in Nursing Students: A Systematic Review and Meta-Analysis. Vol. 2021, Education Research International. Hindawi Limited; 2021.

27. An J, Oh J, Park K. Self-Regulated Learning Strategies for Nursing Students: A Pilot Randomized Controlled Trial. Int J Environ Res Public Health. 2022 Aug 1;19(15).

28. Grech J, Grech J. Nursing students' evaluation of a gamified public health educational webinar: A comparative pilot study. Nurs Open. 2021 Jul 1;8(4):1812–21.

29. Castro MJ, López M, Cao MJ, Castro MF, García S, Frutos M, et al. Impact of educational games on academic outcomes of students in the Degree in Nursing. PLoS One. 2019 Jul 1;14(7).

30. Foss B, Løkken A, Leland A, Stordalen J, Mordt P, Oftedal BF. Digital game-based learning: A supplement for medication calculation drills in nurse education. E-Learning and Digital Media. 2014;11(4):342–9.

31. Blanié A, Amorim MA, Benhamou D. Comparative value of a simulation by gaming and a traditional teaching method to improve clinical reasoning skills necessary to detect patient deterioration: A randomized study in nursing students. BMC Med Educ. 2020 Feb 19;20(1).

32. Chang YS, Hu SH, Kuo SW, Chang KM, Kuo CL, Nguyen T V., et al. Effects of board game play on nursing students' medication knowledge: A randomized controlled trial. Nurse Educ Pract. 2022 Aug 1;63.

33. Integrating Game-based Learning into Undergraduate Nursing Education.

34. Anwer DS, Abdullah SI. Faculty and Students' Perspectives on using Games-Based Learning as A Teaching Strategy in Nursing Education. Journal of University of Raparin. 2023 Mar 29;10(1):147–56.

35. Ordu Y, Çalışkan N. An innovative approach to game-based learning in nursing education: Virtual gaming simulation. Journal of Human Sciences. 2021 Dec 7;18(4):657–64.

36. Abumettleq I, Bayraktar N. Effectiveness of Game-Based Teaching Method on Nursing Students' Knowledge of Enhanced Recovery After Surgery (ERAS). 2023; Available from: https://doi.org/10.21203/rs.3.rs-2444398/v1

37. Calik A, Kapucu S. The Effect of Serious Games for Nursing Students in Clinical Decision-Making Process: A Pilot Randomized Controlled Trial. Games Health J. 2022 Feb 1;11(1):30–7.

38. Wu CS, Chen MF, Hwang HL, LEE BO. Effectiveness of a nursing board games in psychiatric nursing course for undergraduate nursing students: An experimental design. Nurse Educ Pract. 2023 Jul 1;70.

39. Rosa-Castillo A, García-Pañella O, Maestre-Gonzalez E, Pulpón-Segura A, Roselló-Novella A, Solà-Pola M. Gamification on Instagram: Nursing students' degree of satisfaction with and perception of learning in an educational game. Nurse Educ Today. 2022 Nov 1;118.

40. MEŞE S, MEŞE C. Research Trends on Digital Games and Gamification in Nursing Education. Journal of Computer and Education Research. 2022 Dec 21;10(20):734–50.

Appendices

Gantt Chart for PhD Research

YEAR	2022				2023				2024			
MONTH	Jan-Mar	Apr-Jun	July-Sept	Oct-Dec	Jan-Mar	Apr-Jun	July-Sept	Oct-Dec	Jan-Mar	Apr-Jun	July-Sept	Oct-Dec
**ACTIVITY**												
**Enrollment in PhD program**	■	■										
**Identification research area**			■									
**Extensive literature review and finalization of topic**			■									
**Formulation of research strategy, research design & methods**			■									
**Finalization of research synopsis**			■									
**Synopsis presentation at departmental research committee**				■								
**Ethical approval from IEC**				■								
**Approval from doctoral research committee**				■								
**Extensive literature review**					■	■	■	■				
**Implementation of research plan**					■	■	■	■				
**Preparation of scientific reports**							■	■	■			
**Submission of manuscripts to scientific journal**							■	■	■	■	■	
**Final defense**												■
**Graduation**												■
